# Glycolysis and the regulation of glucose transport in *Lactococcus lactis *spp. *lactis *in batch and fed-batch culture

**DOI:** 10.1186/1475-2859-6-16

**Published:** 2007-05-24

**Authors:** Maria Papagianni, Nicholaos Avramidis, George Filiousis

**Affiliations:** 1 Department of Hygiene and Technology of Food of Animal Origin, School of Veterinary Medicine, Aristotle University of Thessaloniki, Thessaloniki 54006, Greece

## Abstract

**Background:**

Despite the fact that many reports deal with glycolysis in *Lactococcus lactis*, there is not much information on the regulation of uptake of glucose itself. The aim of the present work was to investigate the effect of the glucose level on its specific uptake rate.

**Results:**

Studies on aeration levels in pH controlled *L. lactis *spp. *lactis *batch cultures on glucose (55 mM) showed that product formation is extremely homolactic and the highest yield of lactate on glucose is obtained under microaerobic conditions (5% dissolved oxygen). Microaerobic conditions were therefore applied in experiments carried out to investigate the regulation of the uptake of glucose. The tool of glucostat fed-batch culture was employed, in which glucose was added at a rate suitable to maintain a stable concentration throughout the runs with glucose concentration ranging from 13.75 to 555 mM. The glucostat experiments showed that the concentration of glucose influences its specific uptake rate and consequently the glycolytic flux, as well as the fermentation pattern. The highest specific activities of the key glycolytic enzymes PFK, PYK and the LDH were obtained at 55 mM glucose, the area of the highest observed glycolytic flux. Reduction of the glycolytic flux by 55% in the 277 mM glucostat corresponded to an almost identical reduction in PFK activity, indicating a certain controlling influence of this enzyme on the flux, through the glucose effect.

**Conclusion:**

Determination of intracellular metabolites' pools showed that FBP cannot be regarded as a direct regulator of product formation, since almost identical concentrations were obtained at both low (13.75 mM) and high (138 mM) glucose levels, at which neither the glucose uptake rates and the glycolytic flux, nor the fermentation patterns were similar (mixed acids vs homolactic, respectively). Glucostat data showed instead that the control of the flux through the glycolytic pathway under the examined conditions, resides to a large extent in processes outside the pathway, like the ATP consuming reactions and glucose transport. A regulation mechanism is proposed governed by the energy state of the cell by which *L. lactis *can handle the glycolytic flux through the allosteric properties of key enzymes, with PFK having a significant influence on the control.

## Background

Regulation of glycolysis and the shift between different fermentation modes of *Lactococcus lactis *have been extensively studied [1-13]. Key glycolytic enzymes have been characterized and concentrations of glycolytic intermediates in cell extracts have been obtained in many cases since the eighties [11]. However, despite the wealth of available metabolic information for *L. lactis*, the key question of what controls the glycolytic flux in this organism cannot yet be answered unambiguously [11]. When growing on rapidly metabolized sugars, this species shows homolactic metabolism in which more than 90% of metabolized sugar is converted to lactic acid. A deviation from homolactic fermentation is observed under aerobic conditions [14,15] or during the metabolism of galactose [16] or maltose [17]. The mechanisms underlying the shift from homolactic to mixed acid fermentation have been the object of considerable controversy so far and a full explanation has yet to be put forward.

Although sugar metabolism is a central issue in *L. lactis *physiology studies, growth on glucose as the sole carbon source is the case for a relatively small number of studies [3,5,18-20] the majority carried out mainly with lactose. However, Luesink et al. [9,21] showed that growth on glucose resulted in higher activities of the glycolytic key enzymes phosphofructokinase (PFK), pyruvate kinase (PYK), and L-lactate dehydrogenase (LDH), the genes of which form the tricistronic *las *operon. Further on, Even et al. [3], using a novel DNA macroarray technology, showed that several genes of glycolysis were expressed to higher levels on glucose and that genes of the mixed acid pathway were expressed to higher levels on galactose. Also, data given by Even et al. [3] on specific rates of growth, substrate consumption, and product formation (lactate, formate, acetate, and ethanol) during growth of *L. lactis *IL1403 on two different synthetic media (MCD and MS10R) with glucose or galactose as carbon source, show that glucose supports higher growth rates, sugar consumption rates and lactate production rates in both media than galactose.

The above-mentioned studies were carried out under anaerobic conditions. Aeration however, has been shown to strongly influence the cellular content of key enzymes. The negative effect of oxygen on expression of the *pfl *gene (encoding pyruvate formate lyase) is well known [22,23], and the *pfl *gene product is known to be very sensitive to oxygen [23-25]. Expression of the *adh*E gene (encoding alcohol dehydrogenase) is known to be reduced by aeration [26]. The levels of the three key glycolytic enzymes were found to be lower in *L. lactis *cells grown under aerobic conditions [15]. In contrast, the in vitro specific activities of α-acetolactate synthase (ALS) and the pyruvate dehydrogenase (PDH) complex have been reported to increase with aeration [7,27]. Most studies with *L. lactis *have been carried out under totally anaerobic conditions or, in a few cases, under totally aerobic conditions [7,14,27-29]. Intermediate oxygen concentrations have been applied, to the best of our knowledge, only in the works by Jensen et al. [7] and Nordkvist et al. [19] with *L. lactis *ssp. *cremoris*. Both studies were carried out under microaerobic conditions (5 % dissolved oxygen tension-DOT, relative to saturation with air) and with glucose as the sole source of carbon. Comparisons were made at different aeration levels in the work of Nordkvist et al. [19]. The maximum specific growth rate decreased with increasing aeration, while an optimum yield of lactate on glucose was obtained under microaerobic conditions.

Despite the fact that many reports describe the regulatory role of PTS (phosphotransferase system)- and non-PTS-sugars, there is not much information concerning the regulation of uptake of glucose itself and very limited information on growth rates of lactococcal strains on this carbohydrate. Information on the characteristics and properties of transport systems for glucose seem also to be rare. The physiology of *L. lactis *exposed to different concentrations of glucose and the levels of expression of key enzymes in the central pathway of energy production (PFK, PYK, and PDH) is also non-investigated. Recent results reported by Andersen et al. [30] concerning the role of PFK on glycolytic flux in *L. lactis*, show that this enzyme plays an important role since glycolytic and lactate fluxes were decreased proportionally by a twofold reduction of PFK activity. A key role was also attributed to PFK with regard to the glycolytic flux control by Neves et al. [15].

In view of the above and to extend our knowledge of the metabolic behavior of *L. lactis*, we performed batch cultivation experiments with *L. lactis *growing on glucose under different aeration conditions. Having evaluated the effects of aeration, microaerobic conditions were chosen to perform a series of batch fermentations with different initial glucose concentrations and fed-batch fermentations in which glucose was added at a rate suitable to maintain a stable concentration throughout the runs (glucostat). Specific growth rates and the yield on glucose of biomass, lactate, formate, ethanol, acetate and pyruvate were estimated. Specific uptake rates of glucose were determined, as well as the levels of expression of the *las *operon enzymes and the pools of intracellular metabolites for the range of 13.75 to 555 mM glucose concentrations. To further investigate the regulation of glucose uptake by *L. lactis*, the dynamics of glucose transport through the cell membrane were also studied.

## Results

### Effects of different aeration conditions

**Figure 1:**
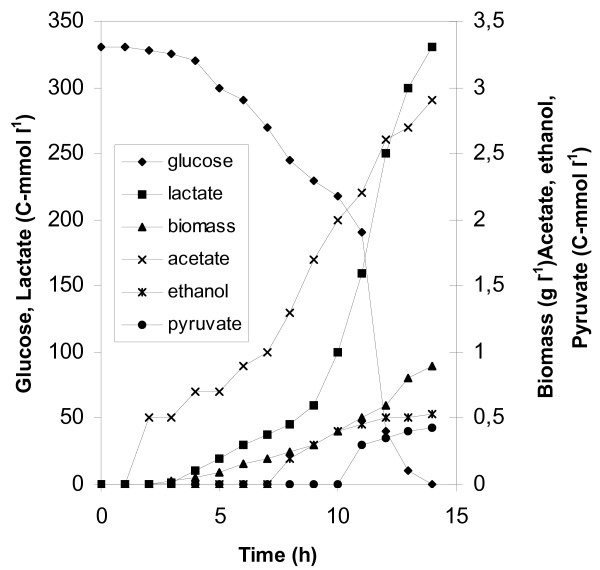
The microaerobic batch fermentation of *L. lactis *ssp. *lactis *LM0230 with 55 mM initial glucose concentration. Product formation and glucose utilization time courses.

Growth of *L. lactis *spp. *lactis *LM0230 on glucose (55 mM) was investigated in pH-controlled batch cultures under the following aeration conditions: anaerobic, microaerobic (5 % DOT), semiaerobic (50 % DOT), and aerobic (>85 % DOT). Under all aeration conditions maximum specific growth rates were high and decreased with increasing aeration (Table 1). The end products accounted for almost 98 % of the converted glucose carbon, and a clear predominance of lactate (>90 %) is obvious under all tested conditions. The yield of biomass on glucose increased with aeration and the highest yield was obtained under fully aerobic conditions. To compare the various glucose carbon recovery data, concentrations are presented as C-mol of product, while the various yield coefficients on glucose as C-mol of product per 100 C-mol of glucose. Under anaerobic conditions the by-products formate, acetate and ethanol were produced at a C-molar ratio of approximately 1:1:1. Under microaerobic, semiaerobic and aerobic conditions the tight constraint on the by-product fluxes was alleviated. Neither formate nor ethanol was formed under aerobic conditions. Under microaerobic conditions product formation was extremely homolactic. Formate was not formed and ethanol was detected in very small amounts. Microaerobic conditions led to a maximum yield of lactate on glucose compared to all other conditions. Figure 1 shows the time course of the batch fermentation with 55 mM glucose performed under microaerobic conditions.

**Table 1:** Maximum specific growth rate and yield coefficients at different aeration conditions*

Aeration conditions	Growth rate (h^-1^)	Yield on glucose of:					
			Products (C-mol of product · 100 C-mol of glucose ^-1^)				
			
		Biomass (g [dry wt] of cells · g of glucose^-1^)	Lactate	Formate	Acetate	Pyruvate	Ethanol

Anaerobic	0.925 ± 0.009	0.162 ± 0.003	92.3 ± 1.3	1.5 ± 0.5	1.5 ± 0.1	0	1.5 ± 0.5
Microaerobic	0.800 ± 0.051	0.151 ± 0.002	96.0 ± 0.5	0	1.1 ± 0.2	0.21 ± 0.02	0.21 ± 0.06
Semiaerobic	0.752 ± 0.007	0.170 ± 0.005	93.2 ± 1.4	0	2.1 ± 0.5	0.10 ± 0.02	0
Aerobic	0.712 ± 0.009	0.182 ± 0.005	91.0 ± 1.5	0	3.2 ± 0.5	0.06 ± 0.03	0

*The values are means ± standard deviations for three independent cultivations. The yield coefficients for biomass, lactate, formate and ethanol were obtained from plots of the amount of accumulated products against the amount of glucose consumed during the exponential phase. Pyruvate was determined at the end of runs and the yield coefficient was calculated on the basis of end concentrations.

### The effect of glucose concentration in microaerobic batch culture

**Figure 2:**
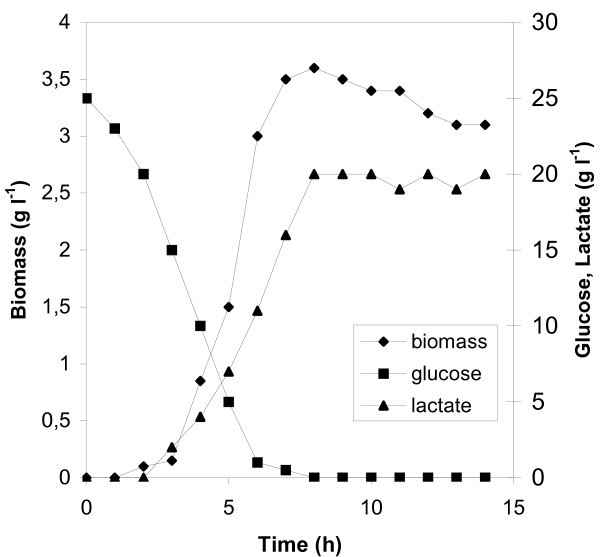
Time course of microaerobic batch fermentation of *L. lactis *ssp. *lactis *LM0230 with 138 mM initial glucose concentration.

**Figure 3:**
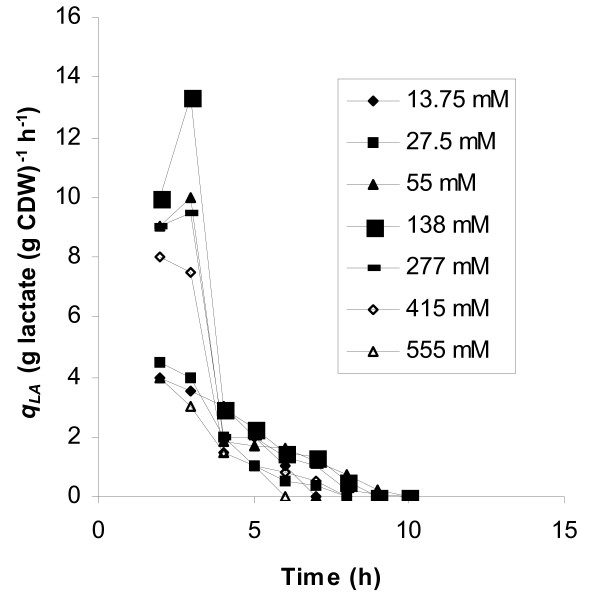
Time courses of specific production rates for lactate in microaerobic batch fermentations with varying initial glucose concentrations.

**Figure 4:**
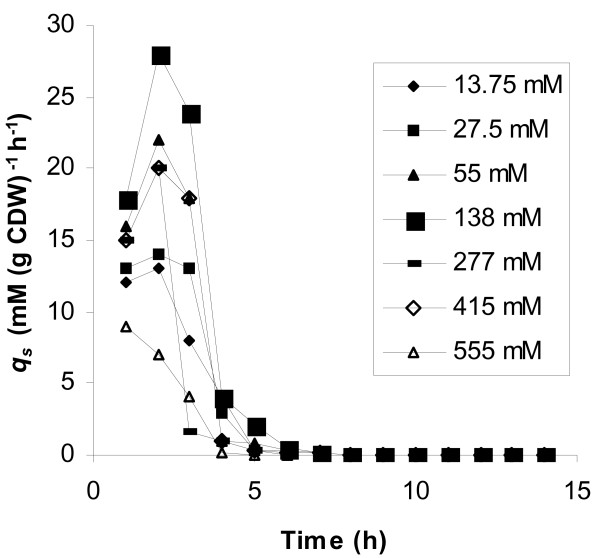
Specific glucose consumption rates during the course of microaerobic batch fermentations performed with different initial glucose concentrations.

Experimental data showed that with respect to the yield on glucose of biomass and lactate, the initial glucose concentration of 138 mM was the optimum. Fig. 2 shows the time course of this fermentation. The maximum specific growth rate measured was 1.22 h^-1^, while the corresponding value at 55 mM initial glucose concentration was 0.8 h^-1 ^(Table 1, Fig. 1). A similar biomass curve with that of Fig. 2 was obtained at 277 mM, with the final biomass concentration not exceeding 3.1 g l^-1^. The highest biomass concentration was obtained at 138 mM initial glucose concentration: 3.6 g l^-1^, while at 55 mM, it was 1 g l^-1^. The maximum concentration of lactic acid obtained in the whole series of fermentations was also noted with 138 mM glucose. Initial glucose concentrations higher that 138 mM resulted in limited glucose consumption and low levels of biomass and lactic acid production. However, lactate was still the main fermentation product since detected concentrations of products of the mixed acids mode were very low (data not shown). Plots of time courses of specific production rates for lactate (*q*_*LA*_) in batch fermentations with different initial glucose concentrations (Fig. 3) were of the same trend for 55, 138 and 277 mM glucose, with the maximum value noted at 3 hours. The highest specific production rates for lactate were obtained at 138 mM glucose at which level biomass concentrations were steadily higher at all time points compared to all other runs. Since, in runs carried out with initial glucose concentrations of 13.75, 27.5, 415, and 555 mM biomass did not exceed 2 g l^-1^, the obtained low specific lactate production rates did not result from any increase in cell mass. Specific glucose consumption rates (uptake rates – *q*_*S*_) are plotted in Fig. 4 according to which, the maximum glucose uptake rate for each initial glucose concentration corresponds to the time-point of the maximum specific production rate for lactate. The initial glucose concentration of 138 mM supported the highest specific growth rate, glucose uptake rate and the highest specific lactate production rate under microaerobic conditions. 13.75 and 27.5 mM initial glucose concentrations led to low yields of lactate and to mixed acid formation.

### The effect of glucose concentration in microaerobic glucostat fed-batch culture

In batch culture the cells experience a constantly changing glucose concentration which undoubtedly affects the outcome of the fermentation. This parameter is excluded when glucose is supplemented at a rate designed to keep its level constant, which is in the present case the glucostat culture. Fed-batch studies operated at constant C-source concentrations were never reported earlier in the literature. The particular mode of glucostat was chosen as the most appropriate tool in evaluations of the glucose level effects in a broad glucose concentration range, since in chemostat cultures only low substrate concentrations can run stably.

In a standard batch culture with 55 mM initial glucose cncentration, the yields of lactic acid on glucose (Y_LA/S_) and NaOH added (Y_LA/NaOH_) were estimated to be 0.922 (*r*^2 ^= 0.995) and 1.721 (*r*^2 ^= 0.995) by linear regression, respectively. Therefore, the ratio of glucose to NaOH can be calculated by the relationship between Y_LA/S _and Y_LA/NaOH_, which can be used in the pH feed-back controlled fed-batch culture. Glucose concentration levels were effectively controlled by the pH feed-back method and maintained well during feeding. Volume differences may have a small effect on fermentation, however care was taken to keep the feeding solution volume as small as 300 ml by adjusting the concentration of the solute to the appropriate levels for each run.

**Figure 5:**
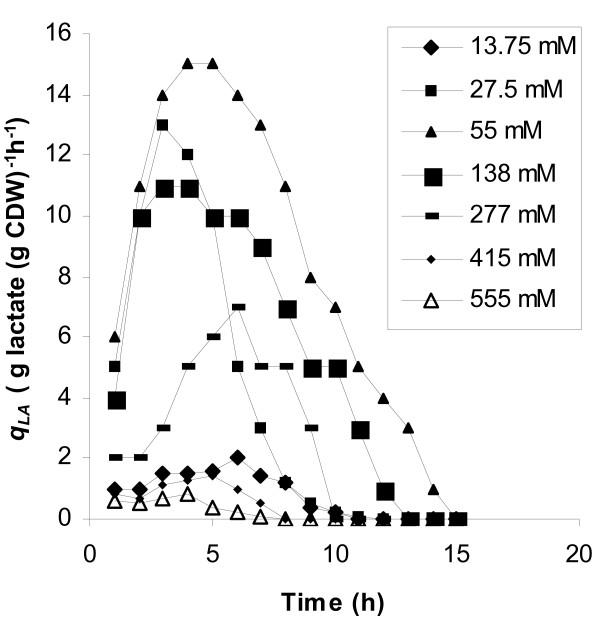
Time courses of specific production rates for lactate at different glucose concentration levels in microaerobic glucostat experiments.

Production phase was longer in glucostat experiments and the overall lactate production was increased compared to batch culture. The influence of the glucose level is very noticeable as both the specific rate of lactate formation and the total concentration reached, increased with the glucose level up to 55 mM. Biomass concentration in the glucostat run of 55 mM glucose reached 4.3 g l^-1 ^and that was the maximum concentration obtained in all tested glucose levels. Inhibition by the substrate was apparent at glucose levels above 138 mM since fermentation rates were reduced and biomass production did not exceed 2 g l^-1^. Fig. 5 shows the plots of specific production rates for lactate. Comparing these plots with the respective plots from batch cultures (Fig. 3) we observe that at glucose concentrations of 138 and 277 mM, specific lactate production rates were even lower than the obtained at 27.5 mM glucose. Maintaining the glucose concentration stable at 27.5 mM during fermentation led to homofermentative product formation, while at 13.75 mM the culture exhibited a shift towards mixed acids metabolism.

**Figure 6:**
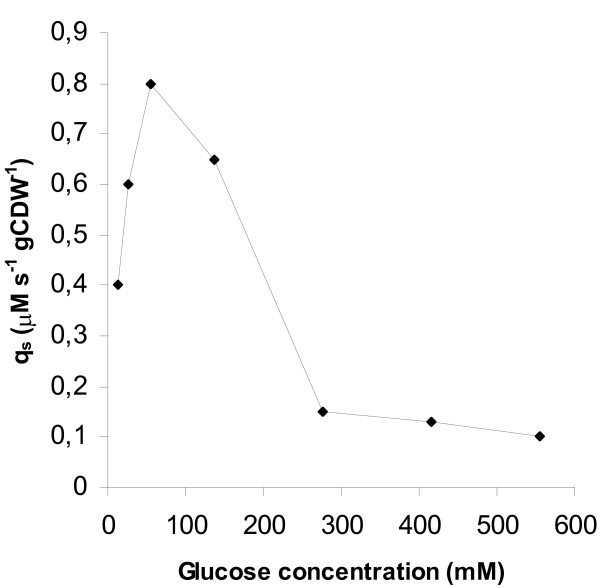
Microaerobic glucostat fermentations. Maximum specific glucose uptake rates obtained at different glucose concentration levels.

Specific substrate consumption rates were estimated (having taken into account the amount of glucose added with feeding) and the maximum values obtained are plotted against glucose concentration in Fig. 6. According to Fig. 6, glucose concentrations beyond 138 mM do not support a high uptake rate. At lower glucose levels, the concentration of glucose has a strong influence on its uptake rate. An optimum glucose level, with regard to its uptake rate, seems to exist and this is 55 mM.

Specific activities of PFK, PYK, and LDH determined in cell extracts from exponential phase cells in glucostat cultures under microaerobic conditions, over a range of glucose concentration levels are shown in Table 2. Activities of the *las *operon genes were different in cells growing at different glucose concentration levels. At 55 mM glucose concentration in glucostat culture, specific activities of PFK, PYK and LDH were the highest. There was a gradual decrease in enzyme specific activities as glucose concentration increased beyond the 55 mM point and in concentrations like 415 and 555 mM, specific activities of all three *las *enzymes were low.

**Table 2:** Specific activities of phosphofructokinase, pyruvate kinase and lactate dehydrogenase in cells (exponential phase) growing in microaerobic glucostat cultures with varying glucose concentration levels

	Sp. Act. of *las *enzyme ^a^		
	
Glucose concentration (mM)	Phosphofructokinase U/OD_600_	Pyruvate kinase U/OD_600_	Lactate dehydrogenase U/OD_600_
13.75	5.00 ± 0.05		
27.50	5.40 ± 0.06	12.52 ± 0.07	21.30 ± 0.08
55.00	7.10 ± 0.05 (4.56)^b^	13.10 ± 0.05 (11.52)^b^	23.72 ± 0.05 (21.30)^b^
138.00	6.00 ± 0.04	11.00 ± 0.08	23.50 ± 0.05
277.00	4.00 ± 0.04	8.72 ± 0.05	11.43 ± 0.07
415.00	3.00 ± 0.05	5.61 ± 0.05	10.70 ± 0.09
555.00	2.11 ± 0.05	5.60 ± 0.07	9.81 ± 0.05

^a^Specific activities (U/OD_600_) are micromoles per minute per cell density. Values are averages of at least three independent measurements.

^b^Values obtained in anaerobic batch cultures with 55 mM initial glucose concentration.

**Figure 7:**
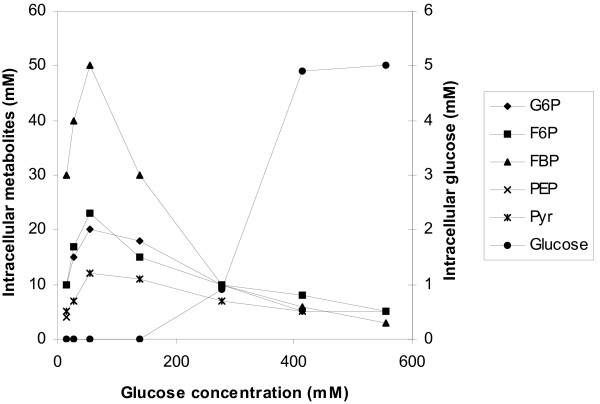
Pools of intracellular metabolites and glucose, determined in cell extracts from samples taken from exponentially growing cells in glucostat cultures with varying glucose concentration levels.

**Figure 8:**
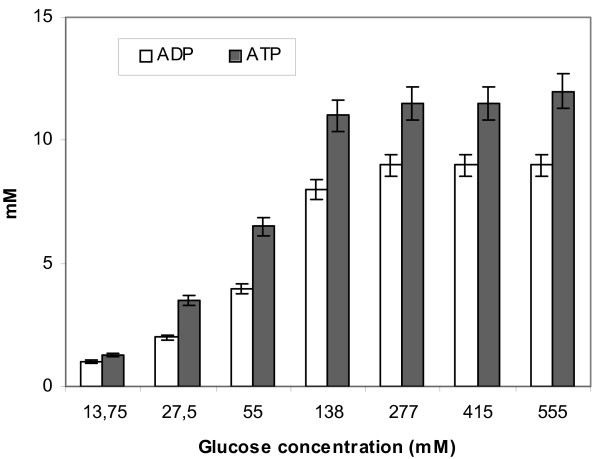
Pools of ADP and ATP, determined in cell extracts from samples taken from exponentially growing cells in glucostat cultures with varying glucose concentration levels.

Intracellular concentrations of glucose-6 phosphate (G6P), fructose-6 phosphate (F6P), fructose-1,6 bisphosphate (FBP), phosphoenolopyruvate (PEP), pyruvate (Pyr), glucose, and the NADH/NAD^+ ^ratio were determined in cell extracts prepared from the same samples as above and are given in Fig. 7. ADP and ATP concentrations were also determined as in Fig. 8. Concentrations of internal metabolites varied with the glucose concentration level. Concentrations of G6F, F6P, FBP and Pyr were the highest at 55 mM. PEP could only me measured accurately in the glucostat run of 13.75 mM glucose. Above 55 mM, the concentrations of G6F, F6P and Pyr decreased gradually, while a sharp fall of FBP concentration was observed. Determination of the NADH/NAD^+ ^ratio showed no direct relationship with the level of glucose. At 13.75 and 138 mM glucose the NADH/NAD^+ ^ratio was found to be 0.07 and 0.06, respectively (results not shown). Intracellular unphosphorylated glucose was detected above 277 mM: at 277 mM its concentration was around 1 mM, while at both 415 and 555 mM, internal glucose concentration reached a 5 mM. According to Fig. 8, at high external glucose concentrations the demands for ATP decrease, since ATP concentrations increased and ADP concentrations decreased.

### Glucose transport studies

Kinetic analysis of the PTS-mediated transport of glucose was performed according to Thompson [12] to investigate the glucose transport capacity of this strain. The initial rates of uptake displayed high-affinity Michaelis-Menten saturation characteristics. This is in accordance to earlier works [12,13] which described the characteristics of glucose transport via the constitutive mannose PTS (Man-PTS). Transformation of the initial rate data according to the method of Eadie-Hofstee yielded the following kinetic parameters: *V*_*max *_= 107 mmol min^-1^g^-1 ^and *K*_*m *_= 2 mM, which are quite different from the reported by Thompson [13] for *S. lactis *ML3. A low affinity carrier appears also to be involved in glucose transport at higher concentrations for which a *V*_*max *_= 278 and *K*_*m *_= 14 mM were estimated. Solving the Michaelis-Menten equation

**Figure 9:**
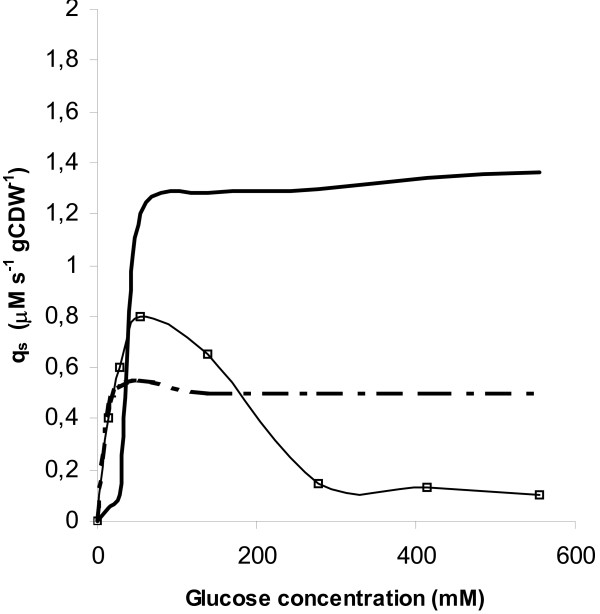
Plots of specific glucose uptake rates corresponding to the low affinity transport system (bold solid line), the high affinity transport system (dashed line) and the experimentally obtained values (thin solid line with points).

V=Vmax⁡⋅SKm+S
 MathType@MTEF@5@5@+=feaafiart1ev1aaatCvAUfKttLearuWrP9MDH5MBPbIqV92AaeXatLxBI9gBaebbnrfifHhDYfgasaacH8akY=wiFfYdH8Gipec8Eeeu0xXdbba9frFj0=OqFfea0dXdd9vqai=hGuQ8kuc9pgc9s8qqaq=dirpe0xb9q8qiLsFr0=vr0=vr0dc8meaabaqaciaacaGaaeqabaqabeGadaaakeaacqWGwbGvcqGH9aqpdaWcaaqaaiabdAfawnaaBaaaleaacyGGTbqBcqGGHbqycqGG4baEaeqaaOGaeyyXICTaem4uamfabaGaem4saS0aaSbaaSqaaiabd2gaTbqabaGccqGHRaWkcqWGtbWuaaaaaa@3CCA@

for the estimated *V*_*max *_and *K*_*m *_values at various glucose concentrations (*S*, mM), the quoted units for *V *were converted to specific uptake rates and plotted along with the experimental derived values for *q*_*s *_in Fig. 9. According to this figure, the experimentally obtained *q*_*s *_is higher than the calculated with the mediated high-affinity transport model. At glucose concentrations 27.5 and 55 mM, glucose is transported by the low-affinity carrier. At even higher glucose levels, accumulation of unphosphorylated glucose inside the cells could be a result of uncontrolled glucose entry by unfascilitated (simple) diffusion.

## Discussion

Results showed that the amount of provided oxygen determines the outcome of fermentation. The equimolar quantities of the by-products formate, acetate, and ethanol produced under anaerobic conditions indicate that the PFL (pyruvate formate-lyase) pathway was the only active pathway of pyruvate metabolism besides LDH, this meaning that there was no flux through the PDH (pyruvate dehydrogenase) enzyme complex. As expected, a different profile was obtained in the presence of oxygen and this can be explained by the activity of NADH oxidases [19]. Since under aerobic conditions neither formate nor ethanol was formed, the fluxes through PFL and alcohol dehydrogenase should be zero, this meaning that the PDH enzyme complex was responsible for the first step in the conversion of pyruvate to acetate. This is in accordance with the work of Jensen et al. [7] who showed that the specific in vitro activities of PDH and NOX (NADH oxidases) increase with aeration. At 5 % DOT microaerobic conditions, product formation was extremely homolactic and the highest yield of lactate on glucose was obtained. Formate was not formed, indicating an inactive PFL enzyme, and the very small amounts of ethanol in the broth should have resulted from activity of the PDH complex. Results of the same trend have been reported by Nordkvist et al. [19] for *L. lactis *ssp. *cremoris *MG1363.

Glucose conversion under microaerobic conditions was complete (Fig. 1) and the maximum specific growth rate was higher compared to that of semiaerobic and aerobic fermentations. The yield of biomass on glucose was the lower obtained within the set of conditions. This must be caused by an increased carbon distribution towards lactate formation since specific glucose uptake rates were high enough in the microaerobic fermentation with an initial glucose concentration of 55 mM (Fig. 4). A quite different situation was obviously obtained from the described by Jensen et al. [7] with *L. lactis *ssp. *cremoris *under microaerobic conditions with a 55 mM initial glucose concentration, where a 5 % DOT in the bioreactor changed the situation of glucose limitation prevalent under anaerobic conditions to one of glucose excess. The reason for the limited glucose consumption was not investigated.

Since microaerobic conditions supported higher specific rates of sugar consumption in the homofermentative mode, we carried out a series of batch fermentations with 5 % DOT, in which the concentration of glucose in the medium differed within the range of 13.75 to 555 mM. These fermentations were used to extract reference points for fed-batch cultivations. Studying the effects of glucose concentration in batch culture, observations unavoidably include the effects of a continuously changing sugar level during the process. This can be avoided with fed-batch culture operated as glucostat, a tool of choice for investigating the effects of glucose concentration levels. A small number of studies [6,13,31,32] have shown that the type and concentration of external sugar affects the concentrations of intermediate metabolites and cofactors and consequently the fermentation productivities. Although these reports are valuable, limitations may exist by the application of anaerobic batch culture only, or by comparisons within a narrow range of concentrations, or even by the use of non-proliferating cells rather than actively fermenting cells. In this study, a series of glucostat experiments were carried out to determine the effects of a stable glucose concentration level, within a broad concentration range, on fermentation rates, key glycolytic enzyme activities, and concentrations of important intracellular metabolites under microaerobic conditions.

In batch culture under microaerobic conditions, the initial glucose concentration of 138 mM supported the highest specific rates for growth, glucose uptake and lactate production. In fed-batch culture, maximal rates obtained by maintaining a continuous 55 mM glucose concentration. In batch culture, specific production rates for lactic acid have an optimum value reached rather early in the fermentation process (Fig. 3). However, the maximum values observed in batch runs occurred when the sugar had fallen to values that according to glucostat data were too low to give significant lactate production. Thus, there must be two aspects to the effect of the sugar. One is the level itself, the other arising from the dynamic situation with cells being exposed to a constantly changing glucose level in the bioreactor. The effect of continuous high glucose concentrations on cell viability may also explain the observed low specific production rates and biomass production levels when glucose exceeded the concentration of 277 mM (Fig. 5).

Glucostat data show that the concentration of glucose is related to its uptake and an optimum uptake rate is observed at 55 mM. Concentrations above 138 mM cannot support a high uptake rate (Fig. 6). The values shown in Fig. 6 obviously represent overall specific uptake rates and include all carbon metabolism of the cell, including excreted products and biosynthesis of cell cytoplasm. It appears from the results that as long as there is a "sink" for the carbon inside the cell, transport can increase in parallel with the sink. If that sink is saturated, then apparent transport will decrease. This is especially true at high glucose concentrations, where glucose appears to be entering the cell by simple diffusion.

Maintaining a continuous low glucose level such as 13.75 mM, specific glucose uptake rates are low and very interestingly, a shift towards mixed acid metabolism takes place. It is known so far for anaerobic culture that homolactic metabolism occurs on substrates supporting rapid growth in which significant amounts of glucose remain in the medium, and a mixed acid fermentation occurs when growth rates are lower and in true carbon-limited chemostats [6,33]. Working the glucostat fed-batch mode under microaerobic conditions, a situation was arranged in which significant amounts of glucose were always present in the fermentation broth with glucose being the substrate supporting the highest fermentation rates [5]. Therefore, the shift from homolactic to mixed acid fermentation could be directly correlated to the glucose uptake rate and consequently to the flux through glycolysis. Indeed, low glycolytic flux has commonly been ascribed to be the cause of mixed acid product formation [3,6,8,34,35]. It has also been shown, that the glycolytic flux governs product formation only when the flux cannot meet the anabolic demand [4]. In response to increased ATP demand the glycolytic flux increases, but if the latter is restricted, mixed acid formation occurs [4]. This is in agreement with the data for ADP and ATP contents of cells presented in Fig. 8 but, the fact that the cells were growing under the glucostat fed-batch mode and glucose was always available but at a fixed, low concentration, points out that apart from the ATP consuming processes, sugar transport should also be involved in the mechanism underlying the phenomena, a fact rather ignored in previously published studies.

The shift from homolactic to mixed acid fermentation in *L. lactis *has been directly correlated to the glycolytic flux, estimated from the specific rates of sugar (glucose, galactose and lactose) consumption, in the work of Garrigues et al. [6]. Under anaerobic conditions, the predominant role of the NADH/NAD^+ ^ratio in controlling the shift was shown, as well as the relationship between glyceraldehyde phosphate dehydrogenase (GAPDH) activity and the NADH/NAD^+ ^ratio. However, under conditions supporting less rapid growth with a diminished flux through glycolysis and a lower NADH/NAD^+ ^ratio, such as growth on lactose and galactose, the major pathway bottleneck was identified at the level of sugar transport rather than GAPDH. The influence of GADPH on glycolysis has been discussed as either strictly controlling [36] or having such a role only when the glycolytic flux is high [6]. Quite different regulatory aspects of glucose metabolism in the presence of oxygen have been reported by Neves et al. [15]. It has been shown that the glycolytic flux was not primarily determined by the level of NADH in the cell, while the main point in that work was the observation that the decrease in the level of PFK activity (40%) was identical to the decrease in the glycolytic flux. A negative effect exerted by oxygen on the glycolytic flux was identified and attributed to depressed PFK activity. Working with another strain (*L. lactis *MG5627), the same group [11] observed a stimulation of glucose consumption under semiaerobic condition, a fact characterized as "apparent discrepancy", which showed that the level of oxygen during growth notably affected the cell metabolic machinery through different effects on gene expression.

In the present study, under conditions of increased glycolytic flux, as in the case of the 55 mM glucostat, the FBP pool (the PFK reaction product) was the largest observed (Fig. 7). It has been proposed [2,37] that FBP has an important role in the regulation of *L. lactis *metabolism acting as an allosteric regulator (activator) of PYK and LDH: high levels of FBP activate PYK and LDH and direct the flux towards lactate production, while low levels of FBP lead to LDH inactivation and inhibition relief of pyruvate-formate lyase by triose phosphates, resulting thus to a shift to mixed acid fermentation. Other researchers have questioned such a direct effect since the intracellular concentrations of FBP in general are sufficient to ensure full activation of LDH [6]. The present work shows that the concentrations of FBP pools and the NADH/NAD^+ ^ratio in the glucostat runs of 13.75 and 138 mM were almost identical, while neither the specific glucose uptake rates (Fig. 6) nor the fermentation patterns were similar. Moreover, much reduced FBP pools at very high glucose concentrations (Fig. 7) suggest that FBP cannot be regarded as a direct regulator of product formation, while they provide an indication of inhibition of PFK activity at such high glucose levels. While the FBP pool level cannot be directly connected to the glycolytic flux and the fermentation pattern, the explanation of the phenomena rather lies in the ATP demand of the cells and the glucose transport capacity of the microorganism.

Specific activities of the key glycolytic enzymes PFK, PYK and the LDH (Table 2), show that expression of the *las *operon genes in microaerobic glucostat cultures was influenced by the glucose level. The 55 mM glucose level supported the highest enzyme activities and this was reflected on the intracellular metabolites pools (Fig. 7). Values obtained in anaerobic batch cultures (Table 2, in parentheses) carried out with 55 mM initial glucose concentration are given for comparison and they are lower compared to those obtained in microaerobic glucostats. Low specific enzyme activities were obtained in the presence of high glucose concentration levels, such as 277 mM. The maximum glycolytic flux of 25.5 mmol g CDW^-1 ^h^-1 ^was observed in the 55 mM glucostat. That was reduced to 14 mmol g CDW^-1 ^h^-1 ^in the 277 mM glucostat, in which fewer ATP consuming processes took place in cells (Fig. 8). The 55% reduction in the glycolytic flux corresponds to a 56% reduction of PFK activity (Table 2). The explanation for the negative influence of elevated glucose levels on the glycolytic flux is likely to lie in part in depressed PFK activity. In addition, the course of FBP concentration over increasing glucose levels and the intracellular accumulation of unphosphorylated glucose (Fig. 7), refer to depressed PFK activity. As mentioned above, a strong influence of PFK on the glycolytic flux was identified in the works by Andersen et al. [30] and Neves et al. [15], in studies with the level of oxygen. A different approach in our work, through the glucose level, demonstrates, and validates, the regulatory role of PFK on glycolytic flux in *L. lactis*.

As with PFKs from *Bacillus stearothermophilus *and *Escherichia coli*, PFK from *L. lactis *is allosterically inactivated by excess ATP [38]. Known inhibitors of PFKs in both prokaryotic and eucaryotic organisms include ATP, citrate and fatty acids, while known activators are ADP, AMP, cAMP, FBP, F6P, NH_4 _and Pi [38-40]. ATP is the major effector and the activators for PFK are better described as deinhibitors of ATP because they reverse the effect of inhibitory concentrations of ATP. PFK is a tetrameric enzyme with two conformational states, R and T, which are in equilibrium. ATP is both a substrate and an allosteric inhibitor of PFK. Each subunit has two binding sites for ATP: a substrate site and an inhibitor site. The substrate site binds ATP equally well in either conformation but the inhibitor site binds ATP almost exclusively in the T state. The other substrate of PFK, F6P, preferentially binds to the R site. Consequently, at high concentrations, ATP acts as a heterotrophic allosteric inhibitor of PFK by binding to the T state, thereby shifting the T-R equilibrium in favour of the T state and thus decreasing PFK's affinity for F6P [40].

The amount of work carried out in the area of glycolysis in *L. lactis *during the last years has been significant. In their very recent review, Neves et al. [11] described the progress made and the further research, which should be pursued for a better understanding of this organism. The need for conducting sugar transport studies was characteristically pointed out. In the present work, glucose uptake rate studies suggested that two uptake systems are involved in the transport of glucose through the cell membrane of *L. lactis *LM0230, a high affinity and low affinity system. Literature information on the transporters of glucose through the cell membrane in *L. lactis *is rather limited [41]. Thompson [12,13] described a high affinity system for glucose transport in *L. lactis *LM3, which corresponds to EII^Man^. The existence of a low affinity system for glucose has not been reported earlier, however evidence for glucose transport by a permease has been reported [13,20]. The trend in glucose transport (Fig. 9) is shown to be saturating when higher glucose concentrations than approximately 40 mM were used. Under conditions in which both carriers are saturated, accumulation of unphosphorylated glucose inside cells could be a result of uncontrolled glucose entry by unfascilitated diffusion, a fact already described with other microorganisms [42]. Accumulation of intracellular glucose and the low levels of phosphorylated sugars under such conditions refer to inhibition of the PFK enzyme.

Thus, we propose a regulation mechanism governed by the energy state of the cell, as this is expressed by the cellular quantities of ADP and ATP, by which *L. lactis *can handle the glycolytic flux under microaerobic conditions. ADP and ATP play central roles in the metabolic network and influence several steps of glycolysis because they are substrates and products of kinases and inhibitors of dehydrogenases. ATP acts as a free-energy donor to drive transport and biosynthesis and it is continuously regenerated from ADP by substrate level phosphorylation. ATP is invested in the upper part of the glycolytic pathway to generate a surplus in the lower part. Moreover, both ATP and ADT serve as precursors in the DNA and RNA synthesis, which has been shown to constitute an approximately 3 and 8% of *L. lactis *cells dry weight, respectively [34]. It has also been shown that intracellular concentrations of ADP and ATP in growing *L. lactis *cells are tightly controlled (homeostatic control) at levels optimal for the cellular reactions [34]. In the present study, under low glucose concentration conditions, provided in glucostat cultures, the glycolytic flux cannot meet the anabolic demand of the cells. There is glucose limitation and consequently energy limitation and the glucose transport capacity of the microorganism is not met, resulting in mixed acids formation. The FBP pool, through LDH and PYK control, does not directly influence product formation since low FBP concentrations were characteristic of both low (13.75 mM) and high (138 mM) glucose concentration levels in glucostat cultures. Therefore, under such conditions, the ATP demand and the glucose transport capacity of the cells are main regulators of the flux. Reversing the situation, by providing constant elevated glucose levels in the glucostat (e.g. above 55 mM), conditions in which glucose transport carriers are saturated, led to excess energy (formation of large intracellular pools of ADP and ATP) which the organism can handle through the allosteric properties of its enzymes. Excess ATP in this case, inhibits PFK activity slowing the glycolytic flux down. It can be suggested here that the extend to which ATP demand controls the glycolytic flux depends on how much excess capacity of glycolysis is present at cells.

## Conclusion

The results in this study suggest that the control of the flux through the pathway of glycolysis resides to a large extent in processes outside the pathway itself, like glucose transport and the ATP consuming reactions. Depending on culture conditions, e.g. dissolved oxygen concentration and glucose concentration levels, the overall flux in *L. lactis *seems to be regulated by the ATP demand through the allosteric properties of key enzymes, with PFK having a significant influence on the control.

## Materials and methods

### Organism and growth conditions

The bacterium used throughout this study was the homofermentative *L. lactis *ssp. *lactis *LM0230 [43]. The strain was stored at -80°C in culture medium supplemented with glycerol (20 %). The medium used for the preparation of inocula and bioreactor cultures was the chemically defined medium (CDM or MCD) described by Otto et al. [44] and modified by Poolman & Konings [45] which contained 47 components, including 18 amino acids and 14 vitamins. The standard medium was supplemented with glucose in the concentration range of 13.75 to 555 mM (2.5 to 100 g l^-1^) as the sole carbon source. Glucose was sterilized separately and added aseptically to the sterile medium in the reactor. For inoculum culture, the medium was supplemented with 0.5 % glucose.

The microorganism was grown in a stirred tank bioreactor -BIOFLO 110, New Brunswick Scientific- with a working volume of 2.0 liters. The reactor was equipped with baffles. The agitation system consisted of two 6-bladed Rushton-type impellers (52 mm), operating at the stirrer speed of 100 rpm. Process temperature was maintained at 30°C and culture pH was maintained at 6.8 by automatic addition of 5 M NaOH. The bioreactor was inoculated with cells from precultures at the end of exponential phase, grown in Erlenmeyer flasks at 30°C under stationary conditions in the medium described above, buffered with 15 g l^-1 ^K_2_HPO_4_. At inoculation time, initial biomass concentration in the reactor was 1 mg l^-1^.

To ensure anaerobic conditions, pure N_2 _was introduced to the reactor prior to inoculation and during the cultivation at a constant in-flow rate of 1 liter of N_2 _per liter of reactor working volume per minute (1 vvm). For aerobic cultivations, the reactor was equipped with a polarographic oxygen sensor (Mettler Toledo, Urdorf, Switzerland). The oxygen electrode was calibrated by sparging the medium with air (DOT, 100 %) and N_2 _(DOT, 0 %); the 100 % saturation value was based on air. Aerobic conditions were obtained by sparging the reactor with air at a rate of 1 vvm to ensure that the DOT was more than 90 % of saturation. For semiaerobic and microarobic experiments the DOT was kept at 50 % and 5 % respectively by sparging the reactor with a mixture of N_2 _and atmospheric air adjusted by using two mass flow controllers, and the DOT was kept constant by feedback regulation of the ratio.

Fermentation volume was 2 liters for batch cultures whereas the initial volume for the glucostat cultures was 1.7 liters and the final 2 liters. Since glucose is mainly converted to lactic acid by the homofermentative *L. lactis*, glucose concentration can be maintained at constant levels by pH feed-back controlled fed-batch culture (controlling the decrease of pH caused by lactic acid accumulation). The method has been described by Lv et al. [46] and employed successfully in studies on the effect of sucrose on nisin production by *L. lactis*. The feed consisted of a glucose (150–300 g l^-1^) and NaOH (140 g l^-1^) solution that was added at a rate of 5 to 10 g l^-1 ^h^-1^, designed to keep the glucose concentration relatively constant, the variation not exceeding 5 %. The concentration of glucose in the feeding solution and the feeding rate were estimated on the basis of the rate of glucose consumption in batch cultures performed with various initial glucose concentrations. The mixture of glucose and NaOH was fed automatically to the vessel by the pH controlled peristaltic pump from the beginning of fermentation. The upper and lower limits for the pH controller were 6.85 and 6.75, respectively. The initial volume of medium in fed-batch cultures contained all other chemicals apart from glucose, in amounts estimated for 1.7 liters of medium. Experiments were carried out at the following glucose levels: 13.75, 27.5, 55, 138, 277, and 555 mM. Batch cultures were performed with the same range of initial glucose concentrations. Samples were taken at inoculation time and at 1-hour intervals until runs' termination at 20 hours. All runs were carried out in triplicate (mean values presented) and repeated if experimental variation exceeded 5 %.

### Analytical methods

#### Biomass

Biomass concentration was monitored spectrophotometrically by measuring the optical density at 600 nm and correlating the optical density with cell dry weight measurements. One unit of optical density at 600 nm was shown to be equivalent to 0.25 g (dry weight) of cells per liter. Cell dry weight was determined by filtering 10 ml of broth through nitrocellulose filters (pore size, 0.2 μm). The filters were tared after having been dried in a microwave oven at 150 W for 15 min. The biomass collected on the filter was washed twice with 10 ml distilled water before being dried and tared as indicated above.

#### Analysis of fermentation products and glucose

Lactic acid concentration was determined with the EnzyPlus D/L Lactic Acid kit by Diffchamb AB (Diffchamb, Sweden). Glucose was determined using the glucose oxidase/peroxidase method of Kunst et al. [47]. Formate, pyruvate, acetate and ethanol concentrations were determined enzymatically using assay kits by Boehringer Mannheim (Mannheim, Germany) (Cat. No. 10979732035 for formate, 10148261035 for acetate, 10176290035 for ethanol) and Sigma-Aldrich (Cat. No. 53006 for pyruvate).

#### Glucose uptake assay

Preparation of *L. lactis *starved cells was done according to Thompson [12]. In the standard transport study procedure, 80 to 100 μl of thick cell suspension was added to 9.8 ml of 0.1 M Tris-(hydroxymethyl)aminomethane maleate buffer (pH 7.0) to obtain a final density of 200 μg (dry weight) of cells per ml. Glucose was added to the system to replicate samples of the starved *Lactococcus *cells in concentrations from 1 μM – 100 mM, and solute accumulation was followed by using the procedures according to Thompson [48]. Uptake assays were carried out at 30°C and no exogenous energy sources were present in transport medium. Initial rates of glucose accumulation (in micromoles per gram [dry weight] of cells per min) were determined after 5 s of incubation. Data were analyzed by computer curve fitting (Leonora 1.0, Oxford Sciences, Oxford, UK). Dry weights were measured on the samples at the end of the experimental period.

#### (iv) Preparation of cell extracts

Bacteria were harvested by centrifugation (16,000 × g, 30 min, 4°C) at an OD_600 _of 1, washed twice with 0.85 % NaCl, and suspended in 20 mM phosphate buffer (pH 6.5) containing 50 mM NaCl, 10 mM MgCl_2_, and 1 mM dithiothreitol [49]. The bacteria were disrupted ultrasonically (20 kHz) at 0°C for 36 cycles of 5 s. Cell debris was removed by centrifugation (13,000 × g, 10 min, 4°C). The protein content of the cell extract was determined by the method of Bradford [50]. For the assay of the phosphoenolopyruvate (PEP)-mannose-phosphotransferase system (PTS), cell debris was not removed because the enzymes are probably linked to the cell membranes [51].

#### Determination of intracellular metabolites

Extracts were prepared from cultures at an OD_600 _of 1, using quenching in hot phenol, according to Andersen et al. [30]. After chloroform extraction, the concentration of sugar-phosphates was measured by recording the increase in NADH fluorescence [13] with the following modification according to Andersen et al. [30]. The buffer contained 50 mM triethanolamine, pH 7.5, instead of imidazolehydrochloride. Glucose-6-phosphate dehydrogenase from yeast was obtained from Boehringer Mannheim, while phosphoglucose-isomerase and fructose bisphosphate aldolase were obtained from Roche Diagnostics GmbH (Mannheim, Germany). Intracellular concentrations of sugar-phosphates were calculated assuming that 1 g (dry weight) corresponds to 1.67 ml of intracellular volume and an OD_600 _of 1 corresponds to 0.25 g (dry weight) per liter.

NAD^+ ^was assayed by using 200 μl of pyrophosphate buffer (250 ml, pH 8.8, containing 12 g of semicarbazide per liter), 10 #956;l of ethanol, 300 μl of extract, 490 μl of H_2_O, and 10 μl of alcohol dehydrogenase (4 mg/ml). The NADH present in the extract was measured in a reaction mixture containing 200 μl of triethanolamine buffer (500 mM, pH 7, containing 15 mM MgSO4 and 4 mM EDTA), 300 μl of extract, 480 μl of H_2_O, 20 μl of pyruvate (200 mM), and 20 μl of LDH (1,000 U/ml) to initiate the pyruvate-dependent oxidation of NADH.

ATP concentrations in cell extracts were measured by a luciferase-luciferin assay mixture (Cat. No. FL-AA 366, Sigma). The sum of the ATP and ADP concentrations was determined after conversion of ADP to ATP by 2 min of incubation in a solution containing triethanolamine buffer (50 mM, pH 7.7), phosphoenolopyruvate (10 μm), MgCl_2 _(2.5 mM) and pyruvate kinase (2 Units). The ADP concentration was calculated by subtracting the ATP concentration from the sum of the ATP and ADP concentrations. Standard curves for ATP, ADP, and mixtures of ATP and ADP corresponded well to each other.

#### Enzyme assays

Enzyme assays were performed at 30°C in a total volume of 1 ml with freshly prepared cell extracts. The formation or consumption of NAD(P)H was determined by measuring the change in the absorbance at 340 nm (ω = 6.22 × 10^3 ^M^-1^.cm^-1^). Values are the means of results from at least two independent duplicate measurements. The blank contained the reaction buffer, the cofactors, and the cell extract without the substrate.

The PTS uptake system for glucose was assayed with a mixture containing 50 mM K_2_PO_4 _buffer (pH 6.8), 5 mM MgCl_2_, 5 mM PEP, 0.5 mM NADH, 4 U of lactate dehydrogenase, and cell extract. The reaction was started by adding 1 mM glucose [52]. Phosphofructokinase (PFK) was assayed by the method previously described by Le Bloas et al. [53] and modified by Even et al. [3] as follows: the reaction mixture for phosphofructokinase contained triethanolamine-HCl buffer (100 mM; pH 7.2), MgCl_2 _(5 mM), KCl (10 mM), ADH (0.3 mM), ATP (5 mM), fructose-1,6P_2 _aldolase (1 U), glycerol-P-dehydrogenase (2 U), triose-P-isomerase (5 U) and fructose-6P (20 mM), which was added to initiate the reaction. Pyruvate kinase (PYK) was assayed according to the method described by Thomas [54] and was optimized as follows: the reaction mixture consisted of Tris-HCl buffer (100 mM; pH 7.2), MnSO_4 _(5 mM), KCl (10 mM), NADH (0.3 mM), lactate dehydrogenase (10 U), GDP (3 mM), and PEP (6 mM). Lactate dehydrogenase (LDH) was assayed by the method optimized by Garrigues et al. [6]. All chemicals were purchased from Sigma at maximum purity.

## Authors' contributions

All authors contributed equally in this work
